# The connection of pollen concentrations and crowd-sourced symptom data: new insights from daily and seasonal symptom load index data from 2013 to 2017 in Vienna

**DOI:** 10.1186/s40413-018-0203-6

**Published:** 2018-10-16

**Authors:** Katharina Bastl, Maximilian Kmenta, Markus Berger, Uwe Berger

**Affiliations:** 1 Aerobiology and Pollen Information Research Unit, Department of Oto-Rhino-Laryngology, Medical University of Vienna, Währinger Gürtel 18-20, 1090 Vienna, Austria; 2 Paracelsus Medizinische Privatuniversität, Strubergasse 21, 5020 Salzburg, Austria

**Keywords:** Symptom data- SLI, Patient’s Hayfever diary, Pollen allergy, Pollen concentrations

## Abstract

**Background:**

Online pollen diaries and mobile applications nowadays allow easy and fast documentation of pollen allergy symptoms. Such crowd-sourced symptom data provides insights into the development and the onset of a pollen allergy. Hitherto studies of the symptom load index (SLI) showed a discrepancy between the SLI and the total pollen amount of a season, but did not analyze the daily data.

**Methods:**

The Patient’s Hayfever Diary (PHD) was used as data pool for symptom data. Symptom data of Vienna (Austria) was chosen as a large and local sample size within the study period of 2013 until 2017. The city was divided into three different areas based on equal population densities and different environmental factors. Correlation factors, regression lines, locally weighted smoothing (LOESS) curves and line plots were calculated to examine the data.

**Results:**

Daily SLI and pollen concentration data correlates well and the progress of the SLI within a pollen season is mirrored by the pollen concentrations. The LOESS curves do not deviate much from the regression line and support the linearity of the symptom-pollen correlation on a daily basis. Seasonal SLI data does not follow the same pattern as the respective seasonal pollen indices. Results did not vary in the three areas within Vienna or when compared with the Eastern region of Austria showing no significant spatial variation of the SLI.

**Discussion:**

Results indicate a linear relationship of the SLI and pollen concentrations/seasonal polllen index (SPIn) on a daily basis for both in general and throughout the season, but not on a seasonal basis. These findings clarify the frequent misinterpretation of the SLI as index that is tightly connected to pollen concentrations, but reflects as well the seasonal variation of the burden of pollen allergy sufferers.

**Conclusion:**

More than just the seasonal pollen index has to be considered when the SLI of a selected pollen season has to be explained. Cross-reactivity to other pollen types, allergen content and air pollution could play a considerable role. The similar behavior of the SLI in Vienna and a whole region indicate the feasibility of a possible symptom forecast in future and justifies the use of a single pollen monitoring station within a city of the size of Vienna.

## Background

Allergies are recognized by the World Allergy Organization (WAO) as a global public health issue [1] – including pollen allergies. Allergic rhinitis makes up a significant proportion of 10–30% of the global population [1] and pollen allergies are still on the rise causing a considerable socioeconomic impact ([2, 3]). One million people of eight million inhabitants are affected by a pollen allergy in Austria [4]. Therefore, pollen information plays a key role in allergen avoidance [5] and is strongly requested by the population [6]. However, the generation of pollen forecasts became more complicated and now has to rely on good fundamental data including symptom data in the best case [7] since pollen measurements alone do not automatically infer the burden that has to be expected from pollen allergy sufferers.

It has been known for some time now, that the relationship of allergic symptoms and allergen/pollen exposure is not a direct one: Ellis et al. [8] showed that the skin test reactivity to ragweed did neither correlate with the rate of symptom development nor the symptom severity. A panel study on grass pollen allergy sufferers revealed that the linear relationship between grass pollen concentrations and grass-sensitized persons is followed by a plateau [9]. One of the early documented causes that could explain at least a part of the observed patterns is the “priming of the end organ” (short: priming effect), which comprises the observed decreased nasal threshold for allergic rhinitis after exposure/challenges and its reversibility [10]. Up to now, the relationship and behavior of symptom patterns and exposure is still not clarified in detail.

Pollen diaries are easy to fill in for users and constitute a useful pool of symptom data nowadays if handled carefully. They provide crowd-sourced data that directly relate to the pollen concentrations and impact on persons concerned. A number of tools, proven valuable, are available (e.g. [11–13]).

The symptom load index (SLI) was developed as an index based on a pollen diary (Patient’s Hayfever diary (PHD); www.pollendiary.com; or in the “Pollen” App) that provides information on the burden of pollen allergy sufferers [14]. As such it is a tool with a direct connection to those who suffer from pollen allergies and provides crucial information. The SLI proved to be a robust index through different pollen seasons or years [15] and is applicable even on a local level [16]. Pollen measurements are needed for the definition of a pollen season and for comparisons with the SLI (e.g. [15, 17]). Thus, the SLI as a number on its own is independent of pollen data and calculated only on symptom data, but is in need for pollen data when a pollen season has to be defined as a prerequisite for a mean SLI in a certain period. Other issues concern the misconception that SLI and pollen data do not correlate. This point will be clarified in detail herein.

It is essential to understand the onset and progress of allergic symptoms for more adequate pollen forecasts [7] and for the development of symptom forecasts. First steps were already taken in this direction: the personalized pollen information classifies users based on their entries and compare them to other users of the same home region which is already reaching beyond a pollen forecast [12]. However, a true symptom forecast is not on the horizon yet and certainly requires a deep understanding of the relationship and behavior of crowd-sourced symptom data.

The goal of this study was to evaluate how the SLI performs/behaves with a focus to (1) repeat a comparison of the felt burden within a certain pollen season (by the means of a mean SLI) with the pollen data of a certain pollen type (by the means of the seasonal pollen integral (SPIn)), (2) to examine if and in which extent correlation between daily SLI and pollen concentrations occur and (3) gain insight into the temporal behavior of the SLI and pollen concentrations throughout a pollen season (e.g., if peaks occur simultaneously).

## Methods

### Pollen data

The pollen data for this study was evaluated with an automatic volumetric pollen and spore trap of the Hirst design [18] situated at the rooftop of the Zentralanstalt für Meteorologie und Geodynamik in Vienna. Pollen data were collected following the minimum recommendations of the European aerobiology community ([19, 20]), also adhered to in the European Aeroallergen Network (EAN; https://ean.polleninfo.eu/Ean/). The following seasons were calculated following the standard season definition in the EAN database starting with 1% of the cumulative annual pollen amount and ending with 95% of the annual pollen amount: alder, birch and grass pollen season. We followed the recently published terminology for aerobiological studies by [21]. Thus, the sum of the average daily (alder, birch or grass) pollen amounts over the defined respective pollen season was calculated as SPIn (formerly also named SPI = seasonal pollen index) and the defined pollen season refers to the MPS (main pollen season in [21]).

### Symptom data

The symptom data used for this study was retrieved from the Patient’s Hayfever Diary (www.pollendiary.com) which is available as well as a mobile application (“Pollen”). Data for analyses were downloaded on 29th of November 2017. Data were selected and retrieved only for districts in Vienna (Austria) for the years 2013 until 2017 since user numbers are especially high from 2013 onwards. More than 50 entries per season allow a statistical analysis of a pollen season in terms of crowd-sourced symptom data [17]. This recommendation was far exceeded by daily mean entries of above 25 (see Area classification). We applied the symptom load index (SLI) described in [14], which comprises in summary the average of entered symptoms (eyes, nose, lungs) and medication intake within a defined time period of a defined user sample. Users are granted anonymity and the services concerned were adapted to fulfill the latest EU regulation on data privacy (Regulation EU 2016/679) resulting in pooling only the absolute minimum of data such as symptom data and an email address in this case. Thus, personal data such as birthday or medical conditions are were not collected. Personal and symptom datasets are saved on separate servers to grant high security to avoid any unauthorized connection between them.

### Area classification

Vienna was divided into three areas to explore the variation of the SLI within a city. The division was based on population density and environmental factors. We established three areas: (1) “Vienna Center” (zip codes: 1010, 1040, 1050, 1060, 1070, 1080, 1090, 1100, 1120, 1150), (2) “Vienna East” (zip codes: 1020, 1030, 1110, 1200, 1210, 1220) and (3) “Vienna West” (zip codes: 1130, 1140, 1160, 1170, 1180, 1190, 1230). Each area within Vienna thus includes dense and less dense populated districts (see [22]). This was cross-checked with the sample size of the PHD and resulted in a very similar sample size for each of the three areas throughout the different pollen seasons and years. A comparable sample size is important to exclude possible influences of an unreliable small dataset. As an example, the mean samples sizes for the year 2017 are noted here for “Vienna Center” (alder/birch/grass pollen season: 26/43/23), “Vienna East” (alder/birch/grass pollen season: 36/60/30) and “Vienna West” (alder/birch/grass pollen season: 38/50/28). The three areas correspond also to areas of a different vegetation influence: “Vienna Center” can be characterized as the most urbanized area with mostly parks and alley trees as green areas. This area is characterized by less grass vegetation (especially natural meadows) and fallow lands (preferred by *Artemisia* and *Ambrosia*). “Vienna East” is influenced by the river Danube and the Danube valley. This area is characterized by alluvial vegetation and taxa preferring proximity to water, e.g., species of the genus *Alnus*, *Populus*, *Salix* and *Fraxinus*. Grass areas and fallow lands are frequent due to the agricultural influence in certain districts (1110, 1210 and 1220). “Vienna West” is influenced by the Vienna forests. Therefore, natural meadows can be found in this area as well as forest elements, such as *Corylus*, *Carpinus*, *Fagus*, *Quercus* and *Pinus*. Fallow lands occur in this area due to the industrial area in certain districts (1130 and 1230). Vienna was chosen for this study due to the high user numbers making it the only location so far possible for a more detailed spatio-temporal analysis of crowd-sourced symptom data.

In addition, the region “Pannonian Lowlands” comprised of Vienna, parts of Lower Austria and Burgenland was included to examine the SLI pattern for a larger area in comparison.

### Statistics

**Fig. 1:**
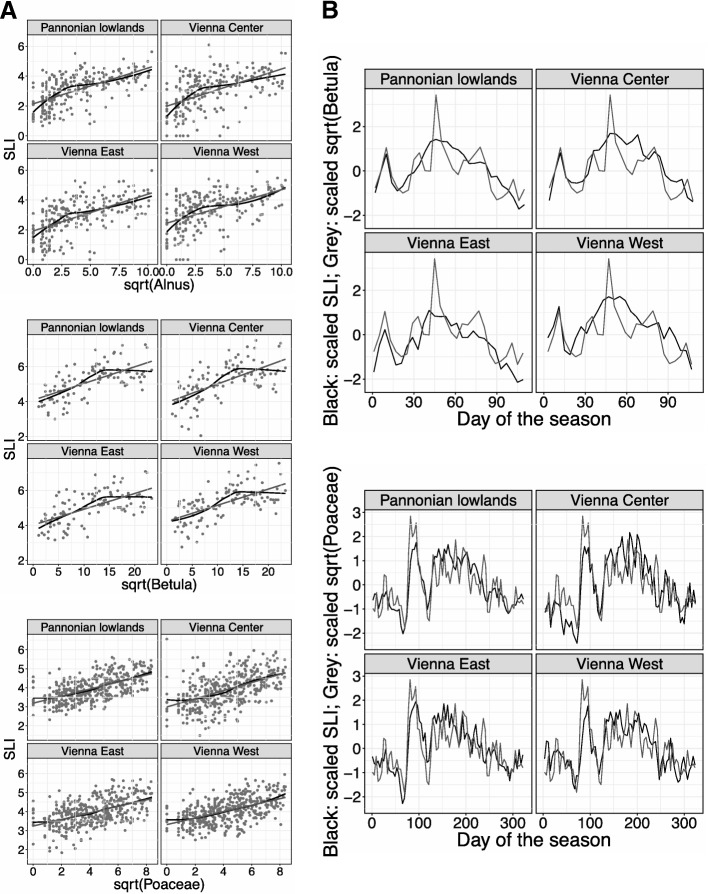
Behavior of the SLI data and the pollen data as square root of the respective aeroallergen is displayed. **a**, Linear regression (grey) and LOESS curve (black) in the three areas within Vienna and the Pannonian Lowlands for the respective pollen seasons (2013–2017) of alder (upper), birch (middle) and grass (lower). Note the similar behavior of the LOESS for all areas/regions within the same pollen season. **b**, Temporal development of square root of the daily pollen concentrations (grey) and the respective SLI data (black). The birch pollen season 2014 (upper) and the grass pollen season 2014 (lower) were chosen as examples to show the similar progress of the datasets. Peaks of the pollen data are reflected in the SLI data

The graphs and computations were performed using the statistical software R 3.4.3 [23]. We used descriptive statistics to explore the behavior of the SLI in comparison to the daily pollen concentrations. Data were analyzed for different regions (see Area classification). The relationship of the SLI and the square root of the daily pollen concentrations is figured in a scatter plot (Fig. 1a). A linear regression line (grey) and a locally weighted smoothing (LOESS) curve (black) were calculated. The linear regression line models the relationship of a dependent variable (SLI) and the explanatory variable (daily pollen concentrations) via the formula y = a + bx. In addition, the correlation factor of the SLI including the *p*-values and significance levels as well as the daily pollen concentrations in the respective pollen season were calculated (see Table 2). LOESS [24] is a modern, non-parametric regression method and was chosen due to its advantages as a flexible and simple tool applied to complex processes without theoretical model. Besides, LOESS finds the curve of best fit without assuming the data have to fit to a certain distribution shape.

The temporal development of the SLI and the daily pollen concentration for specific pollen seasons were explored in a line plot (Fig. 1b). The birch and the grass pollen season 2014 was chosen as an example. The SLI and the square root of the daily pollen concentrations were scaled to mean zero and standard deviation 1 to be comparable. The line chart was chosen to visualize the behavior of the SLI and the daily pollen concentrations over a certain time period (pollen season).

## Results

The SLIs attained in different areas within Vienna show little variation in different years, but the same pollen season (Table 1). In general, the linear relationships and thus the correlation is very similar between the different areas in Vienna and the Pannonian lowlands and is observed due to the parallelism of the linear regression lines (Fig. 1a). Deviations from linearity are shown in the LOESS curve. While the SLI is almost perfectly linear over the whole spectrum of observed grass pollen concentrations, the SLI deviates from the general trend for high birch pollen concentrations (Fig. 1a). The temporal development of the SLI and the square root of the pollen concentrations throughout a pollen season is shown in the line plots (Fig. 1b) and supports the relationship of SLI and pollen concentrations (Table 2). The three pollen seasons analyzed are characterized in the following separately.

**Table 1:** SLI values in different years (2013–2017) through different pollen seasons (alder, birch and grass) are shown for the three selected areas within Vienna (“Vienna East”, “Vienna Center” and “Vienna West”) in combination with their respective average for Vienna

Region	Year	SLI/SPIn alder	SLI/SPIn birch	SLI/SPIn grasses
Vienna Average	2013	2.4/2679	6.4/4517	3.8/3107
Vienna East		2.3	6.2	3.9
Vienna Center		2.4	6.4	3.7
Vienna West		2.6	6.6	3.8
Vienna Average	2014	⇑ 3.6/2883 ⇑	⇓ 5.1/6916 ⇑	⇑ 4.1/2031 ⇓
Vienna East		3.4	4.7	4.1
Vienna Center		3.5	5.3	4.0
Vienna West		4.0	5.3	4.1
Vienna Average	2015	⇓ 2.8/760 ⇓	⇑ 5.6/4992 ⇓	⇑ 4.3/3264 ⇑
Vienna East		2.6	5.6	4.3
Vienna Center		2.5	5.6	4.2
Vienna West		3.2	5.5	4.3
Vienna Average	2016	⇑ 3.9/2685 ⇑	⇓ 5.2/9233 ⇑	⇔ 4.2/2522 ⇓
Vienna East		3.7	5.1	4.2
Vienna Center		4.0	5.3	4.3
Vienna West		4.0	5.2	4.0
Vienna Average	2017	⇑ 4.1/1477 ⇓	⇓ 4.8/3497 ⇓	⇓ 4.0/2517 ⇔
Vienna East		4.0	4.8	3.9
Vienna Center		3.9	4.3	3.9
Vienna West		4.3	5.0	4.4

The SPIn was calculated for the observed time period and is shown for Vienna with the average SLI. The arrows indicate the change of the SLI (left) and the SPIn (right) compared to the previous year. Note the inconsistency between the increase/decrease of SLI and SPIn in most of the cases, especially in the birch and the grass pollen seasons

**Table 2:** The correlation factors including *p*-values (and significance levels) between the daily SLI values of the defined pollen season (alder, birch and grass) in the observed time period (2013–2017) and the daily mean pollen concentrations are displayed for each of the areas in Vienna as well as for a whole region (“Pannonian Lowlands”) in comparison

Corr.-Factor sqrt/*p*-value	SLI alder	SLI birch	SLI grasses
Pannonian Lowlands	69.1/2.02e^− 13^***	69.3/1.90e^− 8^***	69.1/1.85e^− 28^***
Vienna East	59.4/9.15e^− 12^***	66.1/1.68e^− 7^***	62.2/1.77e^− 21^***
Vienna Center	56.4/1.71e^− 10^***	65.9/2.45e^− 7^***	61.5/8.58e^− 21^***
Vienna West	55.3/5.43e^− 10^***	67.9/4.96e^− 8^***	61.9/1.47e^− 21^***

Significance codes: 0 ‘***’ 0.001 ‘**’ 0.01 ‘*’ 0.05 ‘.’ 0.1 “1

### Alder pollen seasons

The mean SLI during the alder pollen season varies from a minimum of 2.4 to a maximum of 4.1 from 2013 to 2017. The three areas within Vienna show a variation from 0.2 to 0.7 between each other in the observed time period. The mean SLI is lowest during the alder pollen season when compared to the birch and grass pollen season. The pattern of increase/decrease in SPIn of alder pollen is consistent from 2014 until 2016, but is interrupted in 2017 (Table 1). Daily alder pollen concentrations correlate with daily symptom data during the alder pollen season. The correlation factors attain values above 50% for the areas within Vienna up to around 70% for the Pannonian Lowlands (Table 2). The behavior of the SLI during the alder pollen seasons 2013–2017 shows the same progress within the three Vienna areas and the Pannonian lowlands (see LOESS curve in Fig. 1a). The data scatters more than for the birch and the grass pollen season and the LOESS curve deviates from the regression line in some extent.

### Birch pollen seasons

The mean SLI during the birch pollen season varies from a minimum of 4.8 to a maximum of 6.4 from 2013 to 2017. The variation between the three areas within Vienna ranges from 0.1 to 0.7 in the observed time period. The mean SLI is highest during the birch pollen season when compared with the alder and grass pollen season. The pattern of increase/decrease in SPIn of birch pollen is inconsistent over most seasons (2014–2016) and only consistent in 2017 (Table 1). Daily birch pollen concentrations correlate with daily symptom data during the birch pollen season. The correlation factors attain values above 60% (Table 2). The behavior of the SLI during the birch pollen seasons 2013–2017 shows the same progress in the three Vienna areas and the Pannonian lowlands (Fig. 1a). The progress within a single birch pollen season (Fig. 1b) shows that the peaks of the square root of the daily mean pollen concentrations is mirrored in the SLI. The three areas in Vienna and the Pannonian lowlands show overall the same pattern as well. Line plots of birch pollen seasons provided similar results.

### Grass pollen seasons

The mean SLI during the grass pollen season varies from a minimum of 3.8 to a maximum of 4.3 from 2013 to 2017. The three areas within Vienna show a variation from 0.1 to 0.5 in the observed time period and thus the lowest variation. The mean SLI attains a value between those of the alder and the birch pollen season. The pattern of increase/decrease in SPIn of grass pollen is mostly inconsistent over most seasons (2014; 2016–2017) with the exception of 2015 (Table 1). Daily grass pollen concentrations correlate with daily symptom data in the grass pollen season. The correlation factors are comparable to those during the birch pollen season and attain as well values above 60% (Table 2). The LOESS for the grass pollen seasons 2013–2017 shows the same pattern within the three areas of Vienna and the Pannonian lowlands (Fig. 1a). The LOESS curve is here most similar to the regression line when compared with the alder and the birch pollen seasons. The progress within a single grass pollen season (Fig. 1b) shows the reflection of the peaks in the square root of the daily mean pollen concentrations in the SLI even better than for the birch pollen season. Only minor differences between the three regions in Vienna and the Pannonian lowlands can be observed (e.g. the double-headed peak during the main peak of the grass pollen season for “Vienna Center” compared to “Vienna East” and “Vienna West”). Line plots of other grass pollen seasons provided similar results.

## Discussion

Daily and seasonal SLIs were analyzed for three different pollen seasons and for three different areas in Vienna to examine the relationship of the SLI and pollen concentrations/SPIn. The SLI data shows a significant correlation with the daily pollen concentrations in general (Table 2) and throughout the season (Fig. 1b). This finding has to be emphasized since the SLI has been misinterpreted since its development [14] in the scientific field and is thwarted by the new and further evidence of its inconsistency as mean value for a pollen season with the respective SPIn of that pollen season (Table 1). The complexity of the results indicate a more or less linear relationship of SLI and pollen concentrations/SPIns on a daily, but not on a seasonal basis. This can be explained by a variety of factors of importance for the development and onset of allergic symptoms.

### Cross-reactivity

Cross-reactivities and other allergic triggers will play a role for individual pollen allergy sufferers. Hazel (*Corylus*) is cross-reactive to alder (*Alnus*) [2] and its occurrence/absence in different seasons may increase or decrease the mean SLI during an alder pollen season. There are several candidates for a possible cross-reactivity during the birch pollen season (all flowering times of possible cross-reactive aeroallergens are based on unpublished long term pollen data from Vienna and personal observation in the following): The hazel and alder pollen season sometimes overlaps with the beginning of the birch pollen season. Beech (*Fagus*) and oak (*Quercus*) flower later during the birch pollen season and are possible triggers for cross-reactivity [2]. Besides, ash (*Fraxinus*) flowers about the same time with birch (*Betula*), but is not closely related. It belongs to the olive family (Oleaceae) and may cause as well pollen allergy during spring time. Poly-sensitized pollen allergy sufferers may react during the analyzed time period to ash and birch pollen. However, the frequency of ash pollen allergy is with 17.7% much lower than birch pollen allergy with 41.7% in Eastern Austria [25]. A number of plants have the potential to irritate pollen allergy sufferers during the grass pollen season such as plantain (*Plantago*), dock (*Rumex*), the nettle family (Urticaceae), the goosefoot family (Amaranthaceae) or mercury (*Mercurialis*) [2]. Grass pollen allergy is the most frequent pollen allergy in Eastern Austria with a frequency of 56.3% [25]. It is noteworthy here to point to the sample size (see Symptom data and Area classification) which is largest for the birch pollen season. This is in accordance with the highest SLIs found in the birch pollen season, despite the lower frequency of birch pollen allergy. User numbers and thus sample size may be connected with a high burden and triggered by the occurrence of severe symptoms. This is reflected as well in pollen information consumption (high user numbers and web site visits) during the birch pollen season [6].

### Allergen content

Significant positive correlations between major aeroallergens such as Bet v 1 and Phl p 5 with the SLI were found for Vienna [26]. It is known that the allergen content may vary from pollen grain to pollen grain [27]. Pollen allergens can be present in the air also as small respirable particles ([28–30]), thus resulting in a different occurrence when compared to pollen concentrations [31]. Allergen content evaluation is highly time and cost consuming. Additionally, not only one major allergen is sufficient to monitor and a range of major and minor allergens are of importance for the immune response (e.g. [32, 33]) besides panallergens such as profilins and polcalcins [34]. Therefore, it remains currently unknown, if a large range of allergens analyzed would explain the symptom burden. Allergen content in any case is a major factor influencing the SLI.

### Air quality and pollution

Air quality in general has an impact on the allergenicity of pollen grains ([35, 36]) and thus the burden of pollen allergy sufferers e.g. the occurrence of atopic diseases (asthmatic bronchitis, hay fever, eczema, sensitization; [37]) and asthma admissions [38]. Allergenicity changes in birch and grass pollen under air pollution such as nitrogen dioxide (NO_2_), ammonia (NH_3_) and ozone (O_3_) and decreases microbial diversity [36]. Majd et al. [35] found that air pollution causes the release of pollen proteins resulting in a higher allergenicity besides a changed shape of the pollen and its tectum. Fine particles shall be mentioned as well, since diesel exhaust carbon particles are able to bind to major allergens under in vitro conditions (e.g. Lol p 1; [39]).

D´Amato et al. [40] summarized the possible relationships between air pollution and allergens as follows: (1) modified allergenicity, (2) interaction with microscopic allergen-carrying particles reaching to the lower airways, (3) inflammatory effect with increased epithelial permeability and (4) adjuvant immunologic effect on IgE synthesis in atopic persons. Therefore, it has to be assumed that air pollution could explain observed SLI patterns in addition.

### Remarks and limitations

The SLI calculations performed herein rely on an excellent sample size and allow to split Vienna in three areas to observe possible spatio-temporal differences. The observation period of five years and the comparison with a whole bio-geographical region (the Pannonian lowlands) adds to the robustness of the outcomes and allows to compare insights in different years and different pollen seasons. A noteworthy impact of the vegetation (see Area classification) could not be observed in the three areas, e.g. the SLI was not highest in “Vienna East”, where the Danube has a strong influence. The results from [14] could be reproduced (seasonal SLI and SPIn do not increase/decrease with each other). This is contrasted by the correlation factors line plots that were produced to show a correlation concerning the daily SLI and daily mean pollen concentrations (Table 2 and Fig. 1a). Moreover, the results do not match with those from Berlin [41] – the only other study that analyzed the spatio-temporal differences of crowd-sourced symptom data within a city to our knowledge. However, the observed differences therein could have occurred due to the much lower sample size in this study in combination with the larger study area.

Care has to be taken concerning the interpretation of the results. Pollen allergy is a complex disease and a range of key factors might play an important role as discussed concerning cross-reactivities, allergenicity and air pollution. Therefore, important limitations are comprised of (1) the SLI is calculated for a certain pollen season, but might be caused also by other aeroallergens and (2) users can not be characterized directly as patients due to the miss of personal information (although the crowd-sourced nature of the data assures significance; e.g., [15] and works also for local phenomena e.g., [16]).

## Conclusions

The in-depth analysis of daily and seasonal SLIs of Vienna revealed that the SLI and pollen concentrations correlate on a daily basis, but not on the seasonal level and that the SLI behavior and pattern do not vary on a local level. In fact, the SLI behavior of a large city like Vienna is more or less the same as for a whole region such as the Pannonian lowlands.

These results lead to far-reaching consequences:

1. a symptom forecast based on crowd-sourced symptom data could be feasible in the future. The development of the SLI could be calculated since the SLI shows a widespread, consistent pattern during the season as shown herein.
2. the overall severity of a season is impacted by additional factors than just the major aeroallergen in the air during this period. The mean SLI during the pollen season usually does not correlation with the SPIn. This should be considered for a range of activities such as pollen forecasting and clinical studies.
3. pollen monitoring stations do not have to be too numerous. Results indicate that a second or more pollen traps in Vienna would not provide any benefit, since the SLI patterns are comparable in the whole city of Vienna. A certain density of pollen monitoring stations across a country/region has of course to be guaranteed and depends on vegetation, topography and other factors.

## Abbreviations

EAN: European Aeroallergen Network (https://ean.polleninfo.eu/Ean)
LOESS: Locally weighted smoothing
PHD: Patient’s Hayfever Diary (www.pollendiary.com)
SLI: Symptom load index
SPIn: Seasonal Pollen Integral
